# Supplementary material for: AlphaFold 2, but not AlphaFold 3, predicts confident but unrealistic β-solenoid structures for repeat proteins
Source: Comput Struct Biotechnol J. 2025 Jan 22;27:467–77. doi: 10.1016/j.csbj.2025.01.016 (PMC11795689; doi:10.1016/j.csbj.2025.01.016)

# Supplementary Material


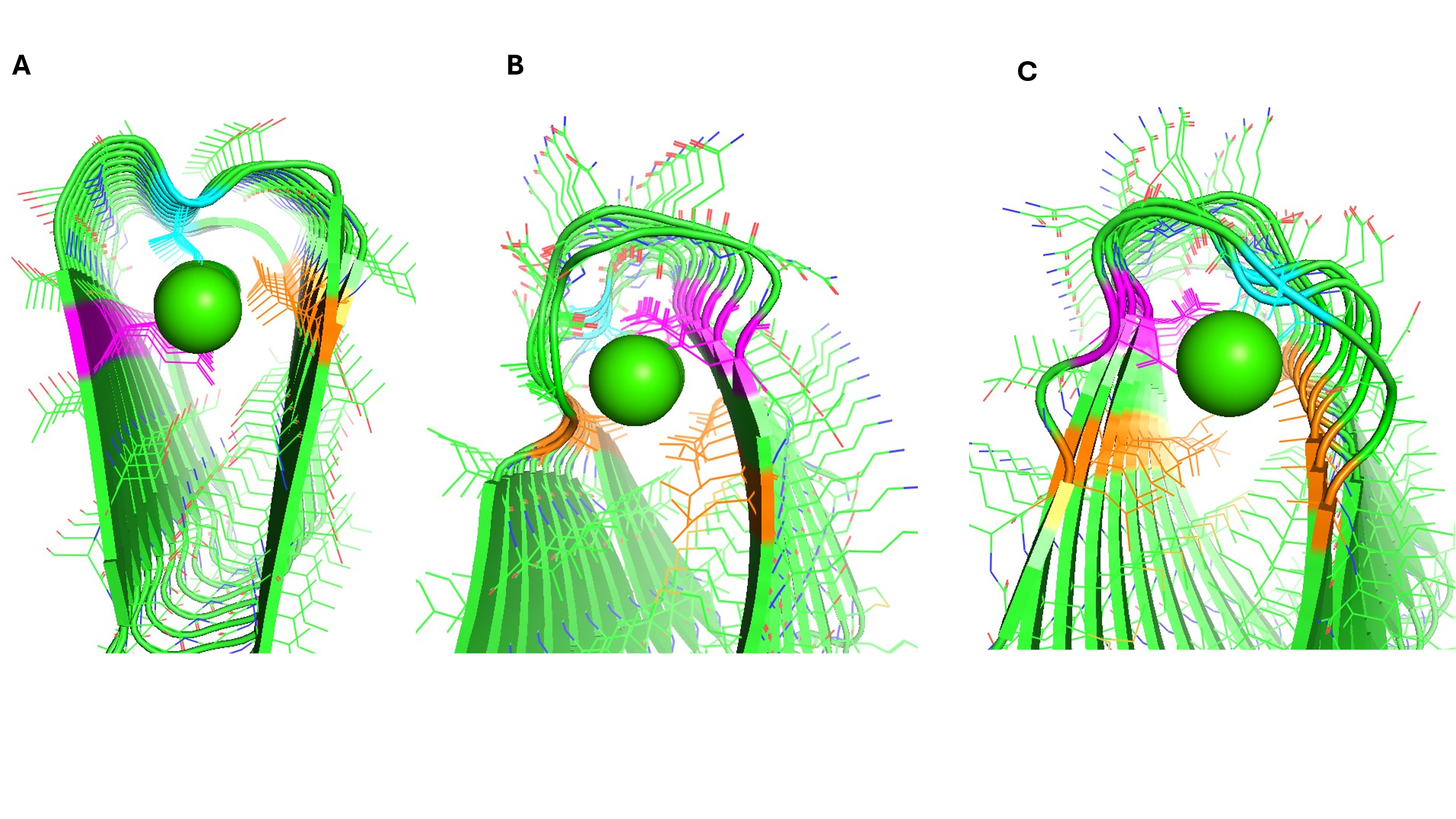


***Figure S1:*** *AF2 models with theoretical internal calcium ion stacks.* ***A)*** *AF2 model of sequence QTSTVIGTIASTETSSSTGI.* ***B)*** *Top view of AF2 model of sequence SMKLTDNQQDAAIIIIVDCFCI.* ***C)*** *Bottom view of AF2 model of sequence SMKLTDNQQDAAIIIIVDCFCI. Internally stacking acidic residues are shown in pink, residues clashing with the calcium ions are shown in blue, and residues that are non-clashing but inappropriate for a calcium binding site are shown in orange. Calcium ions are shown as green spheres. Figure made using PyMOL (pymol.org).*


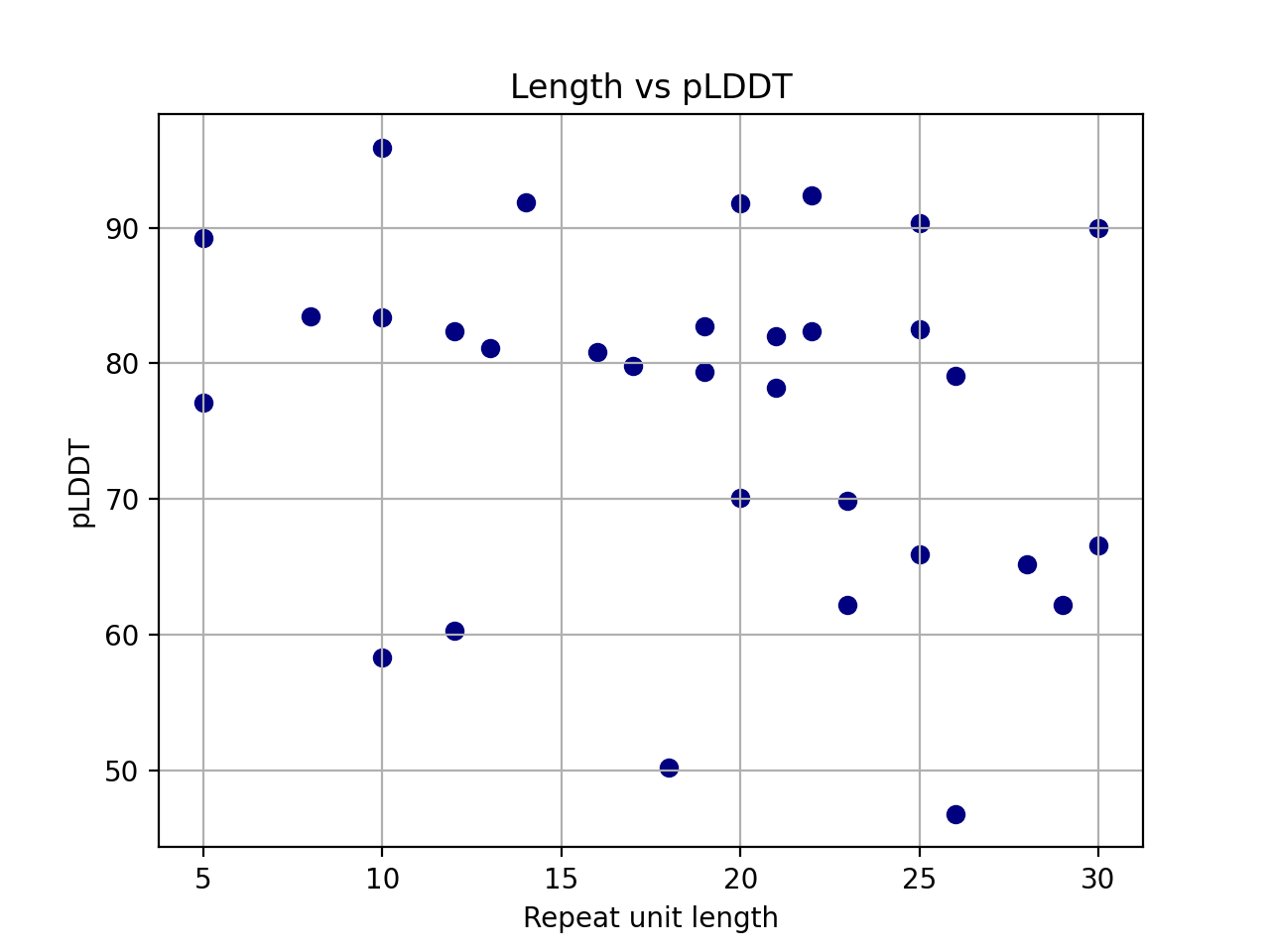


***Figure S2:*** *The repeat unit length of each β-solenoid model plotted against the pLDDT. There are high pLDDT models (>70) across all repeat unit lengths.*

***Figure S3****: Dependence of pLDDT on predicted disorder for AF2 (a, c) and AF3 (b, d), for all structure predictions (a, b) or just for β-solenoid predictions (c, d)*

***Table S2:*** *The representative Pfam family sequence repeats as detected by RADAR showing significant sequence divergence post-duplication.*


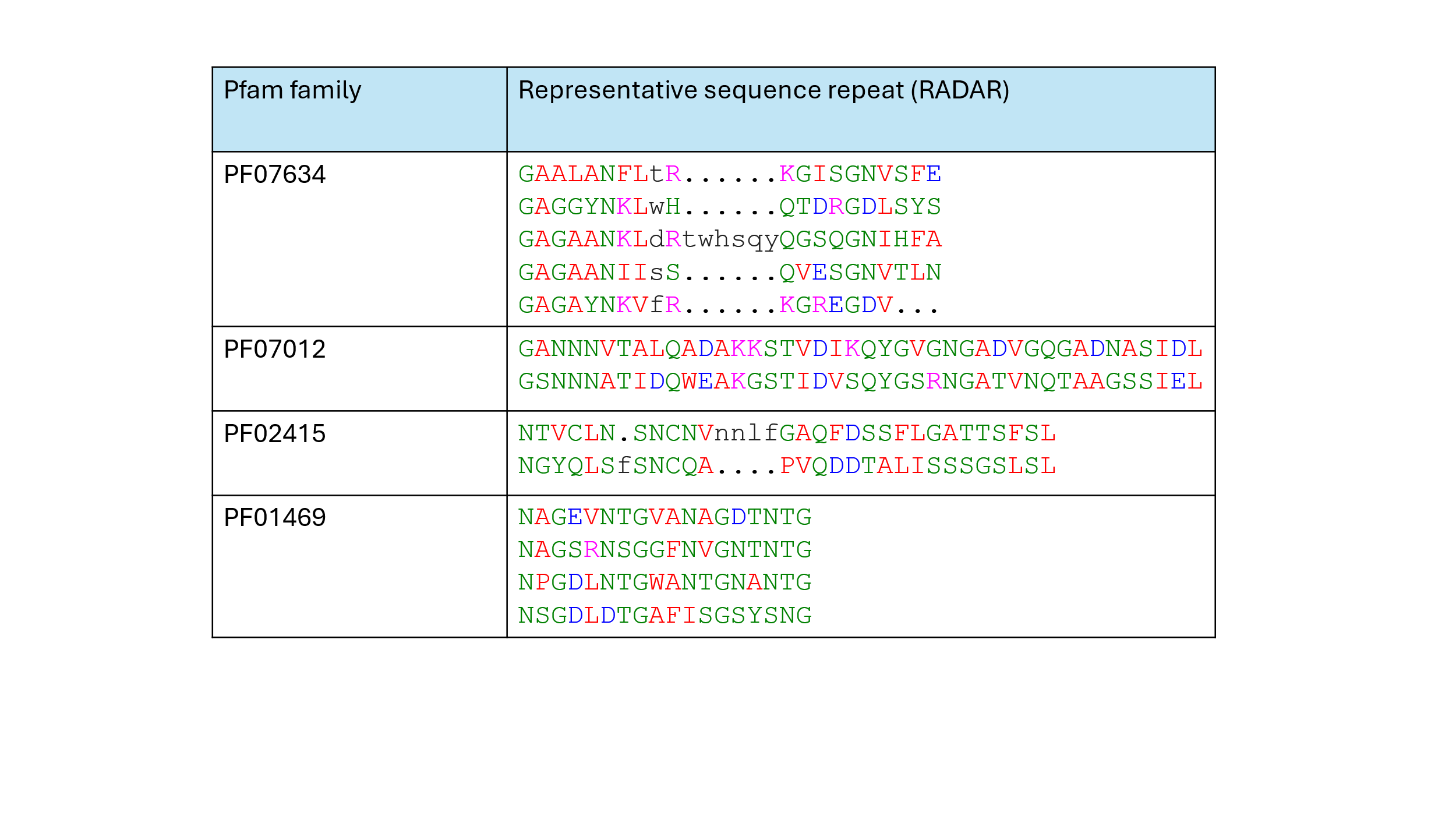

Supplement: Supplementary file 1 — Supplementary material [file mmc1.docx]
